# Incremental mortality associated with nontuberculous mycobacterial lung disease among US Medicare beneficiaries with chronic obstructive pulmonary disease

**DOI:** 10.1186/s12879-023-08689-9

**Published:** 2023-11-01

**Authors:** Ping Wang, Theodore K. Marras, Mariam Hassan, Anjan Chatterjee

**Affiliations:** 1 Insmed Incorporated, 700 US Highway 202/206, Bridgewater, NJ 08807 USA; 2 Medicine, University of Toronto and Toronto Western Hospital, Toronto, ON Canada

**Keywords:** Chronic obstructive pulmonary disease, Incremental mortality, Nontuberculous mycobacterial lung disease, US Medicare

## Abstract

**Background:**

Chronic obstructive pulmonary disease (COPD) is a common comorbidity in patients with nontuberculous mycobacterial lung disease (NTMLD). Both conditions are associated with increased morbidity and mortality, but data are lacking on the additional burden associated with NTMLD among patients with COPD. Thus, the goal of this study was to assess the incremental mortality risk associated with NTMLD among older adults with COPD.

**Methods:**

A retrospective cohort study was conducted using the US Medicare claims database (2010–2017). Patients with preexisting COPD and NTMLD (cases) were matched 1:3 by age and sex with patients with COPD without NTMLD (control patients). Patients were followed up until death or data cutoff (December 31, 2017). Incremental risk of mortality was evaluated by comparing the proportions of death, annualized mortality rate, and mortality hazard rate between cases and control patients using both univariate and multivariate analyses adjusting for age, sex, comorbidities, and COPD severity.

**Results:**

A total of 4,926 cases were matched with 14,778 control patients. In univariate analyses, a higher proportion of cases (vs. control patients) died (41.5% vs. 26.7%; *P* < 0.0001), unadjusted annual mortality rates were higher among cases (158.5 vs. 86.0 deaths/1000 person-years; *P* < 0.0001), and time to death was shorter for cases. This increased mortality risk was also reflected in subsequent multivariate analyses. Patients with COPD and NTMLD were more likely to die (odds ratio [95% CI], 1.39 [1.27–1.51]), had higher mortality rates (rate ratio [95% CI], 1.36 [1.28–1.45]), and had higher hazard of death (hazard ratio [95% CI], 1.37 [1.28–1.46]) than control patients.

**Conclusions:**

The substantial incremental mortality burden associated with NTMLD in patients with COPD highlights the importance of developing interventions targeting this high-risk group and may indicate an unmet need for timely and appropriate management of NTMLD.

**Supplementary Information:**

The online version contains supplementary material available at 10.1186/s12879-023-08689-9.

## Background

Nontuberculous mycobacterial lung disease (NTMLD) is a debilitating condition associated with treatment challenges and high mortality [1–8]. The prevalence of NTMLD has been increasing over the past several decades, both in the United States (US) and worldwide [9–13]. Respiratory diseases such as chronic obstructive pulmonary disease (COPD) are common in patients with NTMLD [5, 6, 14, 15]. Reported estimates of COPD rates in patients with NTMLD have ranged from 28 to 81%; variations in published estimates may be due to differing ages among patient groups [6, 9, 14–18]. In the US, COPD is prevalent, with over 16.4 million diagnosed cases [19]. COPD is associated with serious long-term disability and is consistently among the top 5 causes of death in the US and worldwide [20–22].

NTMLD shares common nonspecific symptoms such as cough and dyspnea with COPD and other respiratory diseases [5, 8, 23–28]; therefore, healthcare providers may have a low index of suspicion for NTMLD [24, 29], leading to delayed diagnosis [24, 26, 28, 30]. An analysis of patients with microbiological and radiographic evidence of active NTMLD found that the most common presenting symptoms were cough, phlegm, fatigue, and dyspnea, and that the average time from symptom onset to NTMLD diagnosis was approximately 5 years [28]. Diagnosis and treatment of NTMLD may be delayed or not prioritized in patients with coexisting lung diseases such as COPD; lack of appropriate management of NTMLD may lead to disease progression and poor clinical outcomes [13, 31, 32].

The clinical burden that NTMLD adds in patients with COPD has not been well characterized. In particular, there are limited data quantifying the incremental mortality burden that NTMLD adds to preexisting COPD [33]. The objective of this study was to assess the incremental mortality burden associated with NTMLD among US Medicare beneficiaries with underlying COPD by comparing their mortality to that of age- and sex-matched control patients with COPD without NTMLD.

## Methods

### Data source

This retrospective cohort study was conducted using 100% beneficiary records in the US Medicare Parts A and B claims database from 2010 to 2017. Medicare is a federal health insurance program for individuals aged 65 years and older, certain younger individuals with disabilities, and individuals with end-stage renal disease [34]. Only patients eligible for Medicare due to age were included in this study.

### Study population

Medicare beneficiaries with NTMLD and preexisting comorbid COPD were identified as meeting the following requirements: (1) the first diagnosis of NTMLD that fulfilled the case definition (see next paragraph) was dated between 2011 and 2016, (2) COPD was diagnosed prior to the first NTMLD diagnosis, and (3) the individual was eligible for Medicare (due to age ≥ 65 years). All eligible patients with both COPD and NTMLD (cases) were matched 1:3 with patients with COPD without NTMLD (control patients) by age and sex.

NTMLD and COPD cases were identified according to previously described methods [12, 35, 36] using *International Classification of Diseases, Ninth Revision, Clinical Modification* (*ICD-9-CM*) codes for claims dated prior to October 1, 2015, and *ICD-10-CM* codes for subsequent claims. A patient with NTMLD was defined as a beneficiary who had ≥ 2 medical encounters with a diagnostic code for NTMLD (**additional file 1**) from either an office visit (diagnostic code must be assigned by a physician), a hospital inpatient stay, or a hospital outpatient visit that were dated ≥ 30 days apart, but within 365 days [12, 35]. A patient with COPD was defined as a beneficiary who had either ≥ 2 ambulatory encounters with a diagnostic code for COPD (**additional file 1**) that were dated ≥ 30 days apart or ≥ 1 hospitalizations with a principal or secondary diagnosis for COPD [36].

To focus on assessment of the incremental burden that NTMLD adds to COPD, we excluded patients with bronchiectasis, as the relationship between bronchiectasis and NTMLD, in terms of both causality and risk, is complex and not fully understood [5, 37, 38]. A patient with bronchiectasis was defined as shown in **additional file 1**.

### Study outcomes

The study population was followed up from index date to death or to the data cutoff date of December 31, 2017. The index date was defined as the date of the first medical claim with diagnosis of NTMLD. The index date of a given case was assigned to the 3 matched control patients. All cases and matched control patients had continuous 1-year pre-index coverage with Medicare Parts A and B. Over the post-index follow-up period, all-cause mortality was compared between patients with COPD with NTMLD and matched control patients with COPD without NTMLD by (1) proportions of patients who died, (2) annual mortality rate, and (3) hazard rate of mortality.

### Statistical analysis

Descriptive analyses of demographic characteristics including age, sex, and race/ethnicity at the index date, as well as of clinical characteristics during the 1-year pre-index period, were conducted for cases with both COPD and NTMLD and matched control patients with COPD without NTMLD.

The incremental mortality associated with NTMLD in patients with pre-existing COPD was assessed with both univariate and multivariate analyses on the mortality over the post-index follow-up period, adjusting for index age, sex, comorbidities, and selected markers of COPD severity during the 1-year pre-index period.

#### Surrogate markers of COPD severity

In clinical practice, the Global Initiative for Chronic Obstructive Lung Disease system is commonly used to assess COPD severity and considers each of the following factors: presence and severity of spirometric abnormality, nature/magnitude of symptoms, history of moderate and severe exacerbations (including hospitalizations), and future risk, as well as presence of comorbidities [39]. For grading of airflow limitation by spirometry, the Global Initiative for Chronic Obstructive Lung Disease uses the post-bronchodilator ratio of forced expiratory volume in 1 s (FEV1) and forced vital capacity (FVC); the Global Initiative for Chronic Obstructive Lung Disease recommends that a post-bronchodilator ratio < 0.70 is indicative of persistent airway obstruction [40]. The Medicare claims data captured some of the standard severity markers such as pulmonary symptoms, comorbidities, and hospitalizations, but other markers such as spirometry findings and the magnitude of symptoms were not available.

Based on clinical experience, we added the following additional surrogate markers of COPD severity: (1) supplemental oxygen use, (2) number of COPD-related hospitalizations per patient, (3) number of COPD-related emergency department (ED) visits per patient, (4) duration from COPD diagnosis to index date, and (5) pulmonary function tests performed (see **additional file 2** for further details).

Univariate analyses of mortality outcomes during follow-up included  (1) the proportion of patients who died, (2) number of deaths per 1,000 person-years, and (3) the Kaplan-Meier survival curves for time to death (patients who were alive at the data cutoff date were censored). Multivariate analyses included logistic regression for estimating the adjusted odds ratio (OR) of death, Poisson regression for the adjusted mortality rate ratio (RR), and Cox proportional hazards model for the adjusted hazard ratio (HR), after controlling for confounding variables.

Categorical variables were presented as the number and percentage of patients. Continuous variables were summarized by mean and standard deviation (SD), median, and quartiles. Statistical tests comparing the case and the control patient groups included McNemar test for categorical variables, Wilcoxon signed-rank test for continuous variables, and log-rank test for time-to-event variables. An α of 0.05 was defined as the threshold for statistical significance.

Statistical analyses were conducted by SAS Enterprise Guide (version 7.15 HF3 [7.100.5.6132; 64-bit]; SAS Institute, Inc).

## Results

### Study population

**Fig. 1:**
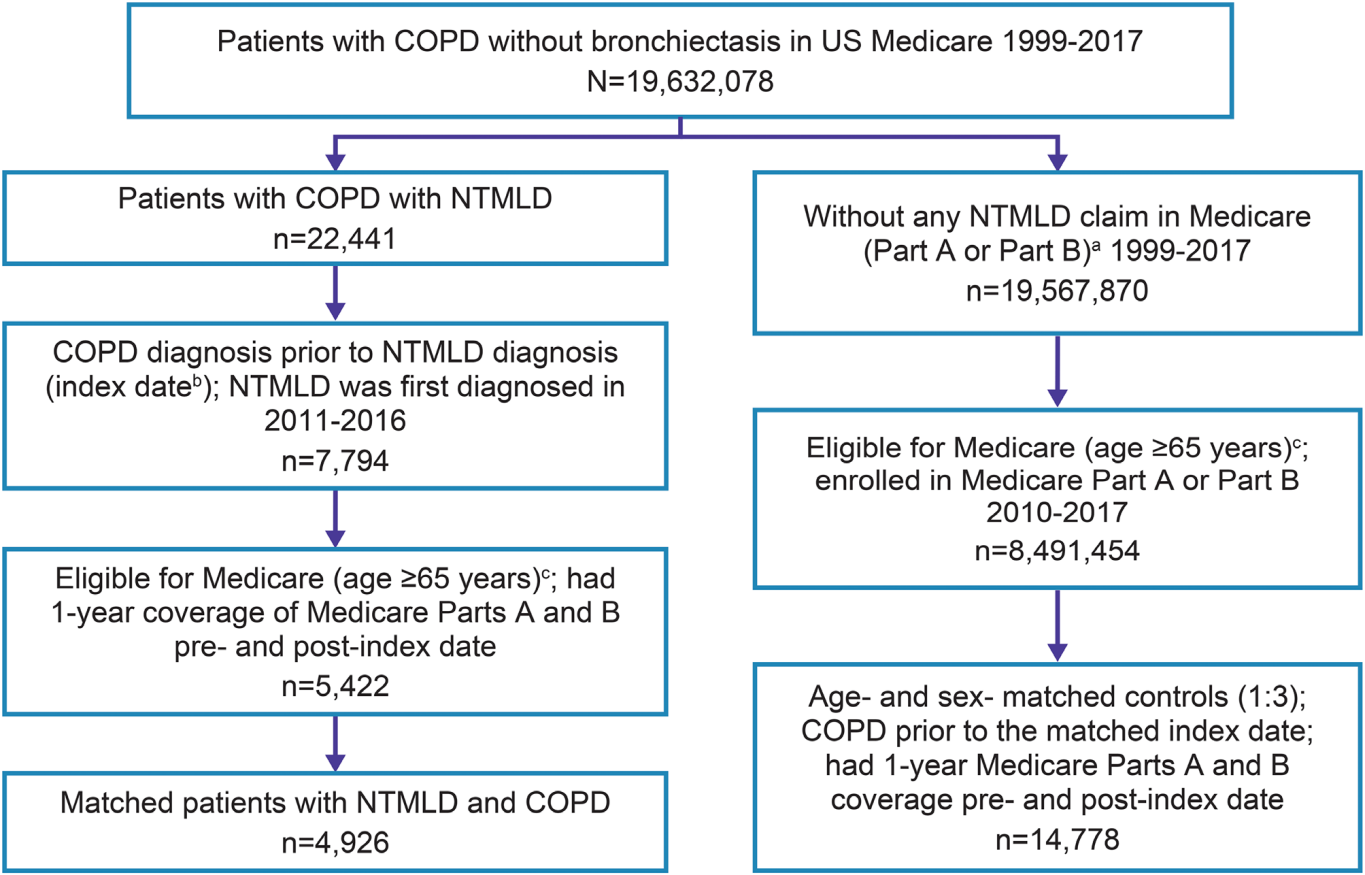
Patient identification from the US Medicare claims database. COPD, chronic obstructive pulmonary disease; DME, durable medical equipment; HHA, home health aide; NTMLD, nontuberculous mycobacterial lung disease; SNF, skilled nursing facility. ^a^Medicare Part A covers inpatient, SNF, HHA, and hospice claims; Medicare Part B covers outpatient, carrier, and DME claims. ^b^Index date: date of first claim for NTMLD (*ICD-9-CM* 031.0 or *ICD-10-CM* A31.0). ^c^Medicare beneficiaries with disabilities or end-stage renal disease were excluded

A total of 4,926 patients met the study case definitions for COPD with NTMLD and were matched with 14,778 control patients with COPD without NTMLD (Fig. 1).

### Demographics and pre-index clinical characteristics

On the index date, patients with COPD with NTMLD (cases) and matched patients with COPD without NTMLD (control patients) had a mean (SD) age of 76.7 (6.7) years, and 54.4% were women (Table 1). The racial/ethnic distribution was similar between groups, with the majority being White (91.4% and 89.0% for cases and control patients, respectively). Clinical characteristics were evaluated during the 1-year pre-index period. Patients with COPD with NTMLD had a higher mean (SD) Charlson Comorbidity Index compared with the matched control patients without NTMLD (2.8 [1.6] vs. 2.2 [1.6], respectively; *P* < 0.0001). Nonpulmonary comorbid conditions that were present in a higher proportion of patients with COPD with NTMLD vs. control patients included being underweight or having abnormal weight loss (21.9% vs. 6.0%; *P* < 0.0001), rheumatoid arthritis (6.9% vs. 4.3%; *P* < 0.0001) and gastroesophageal reflux disease (41.1% vs. 29.0%; *P* < 0.0001). Nonpulmonary comorbid conditions that were present in a lower proportion of patients with COPD with NTMLD vs. control patients included diabetes (25.7% vs. 35.4%; *P* < 0.0001) and overweight and obesity (7.9% vs. 11.6%; *P* < 0.0001). Nearly all of the pulmonary symptoms and conditions listed in Table 1 occurred in a higher proportion of patients with COPD with NTMLD vs. control patients, including cough (63.0% vs. 24.4%; *P* < 0.0001), dyspnea (72.7% vs. 37.6%; *P* < 0.0001), emphysema (39.0% vs. 10.3%; *P* < 0.0001), and pneumonia (58.5% vs. 12.2%; *P* < 0.0001). The diagnostic codes used for identifying comorbidities can be found in **additional file 3**.

**Table 1:** Patient Demographics and Clinical Characteristics During the 1-Year Pre-index Period

Demographic and Clinical Characteristics	COPD With NTMLD (n = 4,926)	COPD Without NTMLD (n = 14,778)	*P* Value^a^
Age at index date, years			
Mean (SD)	76.7 (6.7)	76.7 (6.7)	
Median (Q1, Q3)	76 (71, 81)	76 (71, 81)	
Female, n (%)	2,680 (54.4)	8,040 (54.4)	
Race/ethnicity, n (%)			
White	4,503 (91.4)	13,151 (89.0)	
Black	173 (3.5)	938 (6.4)	
Asian	102 (2.1)	214 (1.4)	
Hispanic	46 (0.9)	202 (1.4)	
North American Native	20 (0.4)	76 (0.5)	
Other or unknown	82 (1.7)	197 (1.3)	
Charlson Comorbidity Index, mean (SD)	2.8 (1.6)	2.2 (1.6)	< 0.0001
Nonpulmonary comorbid conditions, n (%)			
All cardiovascular diseases^b^	4,564 (92.7)	13,302 (90.0)	< 0.0001
Hypertension^b^	3,924 (79.7)	12,079 (81.7)	< 0.0001
Gastroesophageal reflux disease^b^	2,025 (41.1)	4,289 (29.0)	< 0.0001
Malnutrition^b^	1,284 (26.1)	3,096 (21.0)	< 0.0001
Diabetes mellitus^b^	1,264 (25.7)	5,226 (35.4)	< 0.0001
All cancers, excluding lung cancer^b^	1,150 (23.4)	2,355 (15.9)	< 0.0001
Underweight or abnormal weight loss	1,077 (21.9)	886 (6.0)	< 0.0001
Chronic kidney disease^b^	890 (18.1)	2,580 (17.5)	< 0.0001
Dementia^b^	535 (10.9)	2,110 (14.3)	< 0.0001
Overweight and obesity^b^	388 (7.9)	1,720 (11.6)	< 0.0001
Rheumatoid arthritis^b^	341 (6.9)	641 (4.3)	< 0.0001
Crohn’s disease	47 (1.0)	85 (0.6)	0.0009
Systemic lupus erythematosus	46 (0.9)	81 (0.5)	0.002
Ulcerative colitis	45 (0.9)	105 (0.7)	< 0.0001
Chronic viral hepatitis	40 (0.8)	56 (0.4)	0.10
Moderate or severe liver disease^b^	36 (0.7)	66 (0.4)	0.003
Transplant of kidney, heart, or liver^b^	32 (0.6)	29 (0.2)	0.70
Human immunodeficiency virus	23 (0.5)	17 (0.1)	0.34
Multiple sclerosis	9 (0.0)	31 (0.2)	0.0005
Pulmonary symptoms, n (%)			
Dyspnea	3,582 (72.7)	5,552 (37.6)	< 0.0001
Cough	3,103 (63.0)	3,608 (24.4)	< 0.0001
Hemoptysis	635 (12.9)	198 (1.3)	< 0.0001
Pulmonary comorbidities, n (%)			
Pneumonia	2,884 (58.6)	1,804 (12.2)	< 0.0001
Simple and mucopurulent chronic bronchitis^b^	2,019 (41.0)	2,424 (16.4)	< 0.0001
Emphysema	1,919 (39.0)	1,525 (10.3)	< 0.0001
Smoking history	1,887 (38.3)	2,585 (17.5)	< 0.0001
Idiopathic interstitial lung disease^b^	1,321 (26.8)	566 (3.8)	< 0.0001
Asthma^b^	1,307 (26.5)	2,552 (17.3)	< 0.0001
Malignant neoplasm of bronchus and lung^b^	797 (16.2)	458 (3.1)	< 0.0001
Pulmonary tuberculosis^b^	505 (10.3)	18 (0.12)	< 0.0001
Idiopathic pulmonary fibrosis^b^	126 (2.6)	52 (0.35)	< 0.0001
Lung transplant	37 (0.75)	5 (0.03)	< 0.0001
Cystic fibrosis with pulmonary manifestations	2 (0.0)	0 (0.0)	

COPD, chronic obstructive pulmonary disease; NTMLD, nontuberculous mycobacterial lung disease; Q, quartile; SD, standard deviation

^a^*P* values were based on a McNemar test for categorical variables and Wilcoxon signed rank test for continuous variables

^b^Comorbidities included in the multivariate analysis

#### Pre-index COPD severity

During the 1-year pre-index period, patients with COPD with NTMLD demonstrated more severe COPD (vs. control patients with COPD without NTMLD) based on our severity markers, including the mean (SD) numbers of COPD-related hospitalizations (0.2 [0.5] vs. 0.1 [0.3]; *P* < 0.0001) and COPD-related ED visits (0.2 [0.9] vs. 0.1 [0.7]; *P* < 0.0001), as well as the proportion of patients with use of supplemental oxygen (29.6% vs. 14.5%; *P* < 0.0001) and with pulmonary function tests performed (49.7% vs. 17.6%; *P* = 0.03) (Table 2). When the pre-index measures of COPD severity were stratified by patient survival status, all measures were higher for patients with COPD with NTMLD compared with control patients for both the alive and deceased subgroups (Table 2). Among patients with both COPD and NTMLD, the following severity markers were notably higher for patients who died during the follow-up period vs. those who were alive at the end of the follow-up period: mean (SD) of COPD-related hospitalizations (0.22 [0.6] vs. 0.11 [0.41]) and COPD-related ED visits (0.33 [1.03] vs. 0.18 [0.68]) and the proportion of patients with supplemental oxygen use (41.9% vs. 20.9%).

**Table 2:** Markers of COPD Severity During the 1-Year Pre-Index Period

Markers of COPD Severity	COPD With NTMLD	COPD Without NTMLD	*P* Value^a^
Number of COPD-related hospitalizations per patient^b^			
All, n	4,926	14,778	
Mean (SD)	0.2 (0.5)	0.1 (0.3)	< 0.0001
Alive at the end of follow-up, n	2,884	10,836	
Mean (SD)	0.11 (0.41)	0.04 (0.22)	< 0.0001
Deceased at the end of follow-up, n	2,042	3,942	
Mean (SD)	0.22 (0.60)	0.11 (0.39)	< 0.0001
Number of COPD-related ED visits per patient^b^			
All, n	4,926	14,778	
Mean (SD)	0.2 (0.9)	0.1 (0.7)	< 0.0001
Alive at the end of follow-up, n	2,884	10,836	
Mean (SD)	0.18 (0.68)	0.09 (0.66)	< 0.0001
Deceased at the end of follow-up, n	2,042	3,942	
Mean (SD)	0.33 (1.03)	0.21 (0.85)	< 0.0001
Years from COPD diagnosis to index date^b^			
All, n	4,926	14,778	
Mean (SD)	6.0 (4.5)	6.3 (4.2)	< 0.0001
Median (Q1, Q3)	5.23 (2.12, 9.50)	5.65 (2.67, 9.37)	
Alive at the end of follow-up, n	2,884	10,836	
Mean (SD)	5.57 (4.43)	5.98 (4.13)	< 0.0001
Median (Q1, Q3)	4.62 (1.74, 8.71)	5.27 (2.48, 8.90)	
Deceased at the end of follow-up, n	2,042	3,942	
Mean (SD)	6.60 (4.39)	7.01 (4.25)	< 0.0001
Median (Q1, Q3)	6.10 (2.84, 10.23)	6.84 (3.34, 10.37)	
Proportion of patients with supplemental oxygen^b^			
All, n	4,926	14,778	
n (%)	1,459 (29.6)	2,136 (14.5)	< 0.0001
Alive at the end of follow-up, n	2,884	10,836	
n (%)	604 (20.94)	1,176 (10.85)	< 0.0001
Deceased at the end of follow-up, n	2,042	3,942	
n (%)	855 (41.87)	960 (24.35)	< 0.0001
Proportion of patients with pulmonary function test performed			
All, n	4,926	14,778	
n (%)	2,448 (49.7)	2,606 (17.6)	0.03
Alive at the end of follow-up, n	2,884	10,836	
n (%)	1,551 (53.78)	1,982 (18.29)	< 0.0001
Deceased at the end of follow-up, n	2,042	3,942	
n (%)	897 (43.93)	624 (15.83)	< 0.0001

COPD, chronic obstructive pulmonary disease; ED, emergency department; NTMLD, nontuberculous mycobacterial lung disease; Q, quartile; SD, standard deviation

^a^*P* values were based on a McNemar test for categorical variables and Wilcoxon signed rank test for continuous variables

^b^Markers of COPD severity included in the multivariate analyses

### Mortality associated with NTMLD

**Fig. 2:**
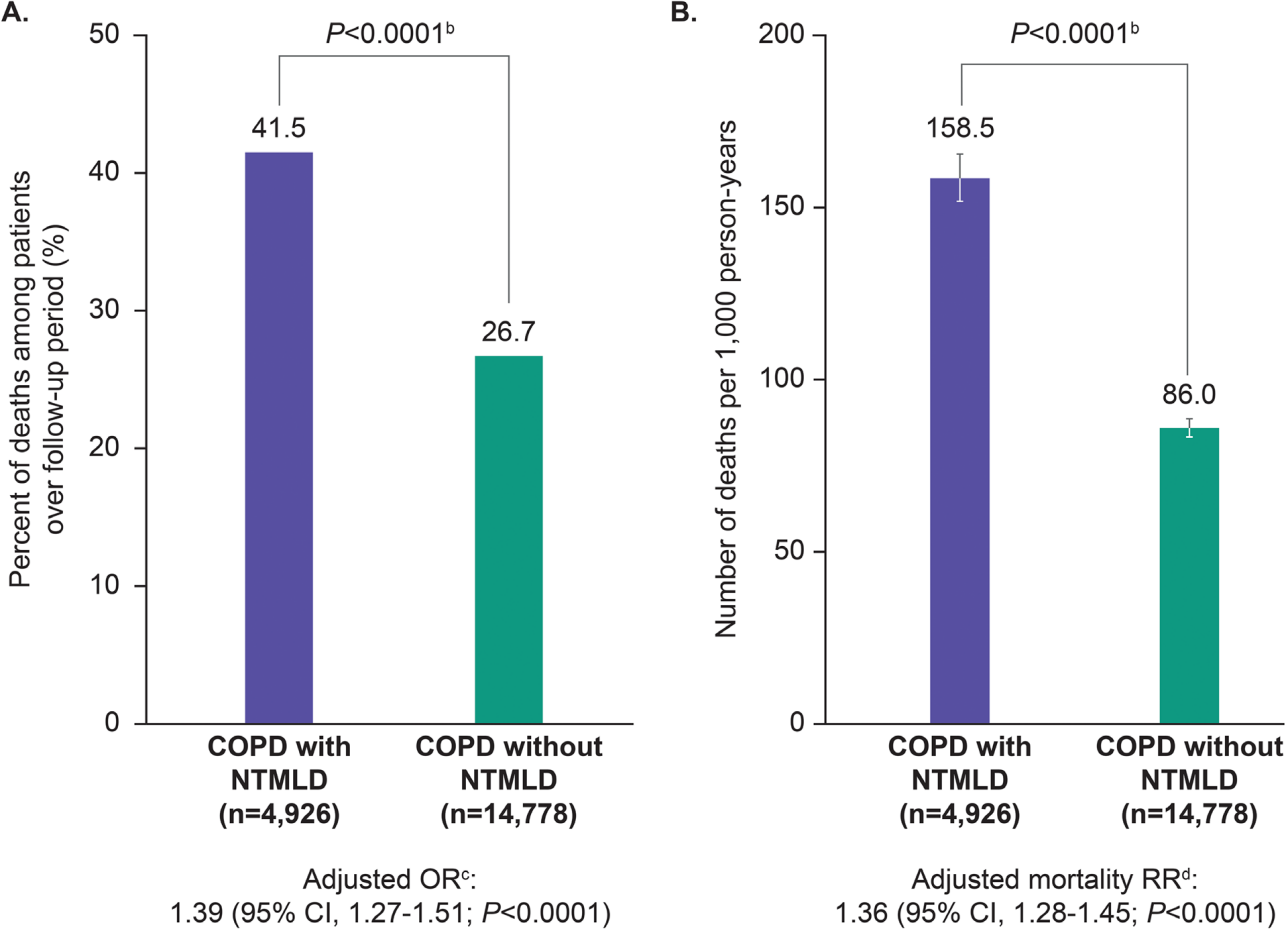
Increased mortality associated with NTMLD: proportion of deaths (**A**); unadjusted annual mortality rate (**B**).^a^ COPD, chronic obstructive pulmonary disease; NTMLD, nontuberculous mycobacterial lung disease; OR, odds ratio; RR, rate ratio. ^a^Error bars represent the 95% confidence interval. ^b^*P* value was based on McNemar test for categorical variables and Wilcoxon signed rank test for continuous variables. ^c^Adjusted OR associated with NTMLD was derived from multivariate logistic regression analysis controlling for age, sex, comorbidities, and COPD severity during the 1-year pre-index period. ^d^Adjusted RR associated with NTMLD was derived from multivariate Poisson regression analysis controlling for age, sex, comorbidities, and COPD severity during the 1-year pre-index period

The mean (SD) follow-up duration from index date to death or data cutoff was 2.6 (1.7) years for patients with COPD and NTMLD and 3.1 (1.8) years for control patients with COPD without NTMLD. A higher proportion of patients with both COPD and NTMLD died during follow-up than patients with COPD without NTMLD (41.5% vs. 26.7%; *P* < 0.0001; Fig. 2A). After multivariate logistic regression analysis controlling for confounding factors, including age, sex, selected comorbidities (Table 1), and markers of COPD severity during the 1-year pre-index period, patients with both COPD and NTMLD were 39% more likely to die than patients with COPD without NTMLD (OR, 1.39; 95% confidence interval [CI], 1.27–1.51; *P* < 0.0001).

Unadjusted annual mortality rates were higher among patients with both COPD and NTMLD vs. patients with COPD without NTMLD (158.5 vs. 86.0 deaths per 1,000 person-years; *P* < 0.0001; Fig. 2B). Similarly, in multivariate Poisson regression analysis controlling for confounding factors, including age, sex, selected comorbidities, and markers of COPD severity during the 1-year pre-index period, patients with both COPD and NTMLD had 36% higher mortality rate than control patients with COPD without NTMLD (RR, 1.36; 95% CI, 1.28–1.45; *P* < 0.0001).

**Fig. 3:**
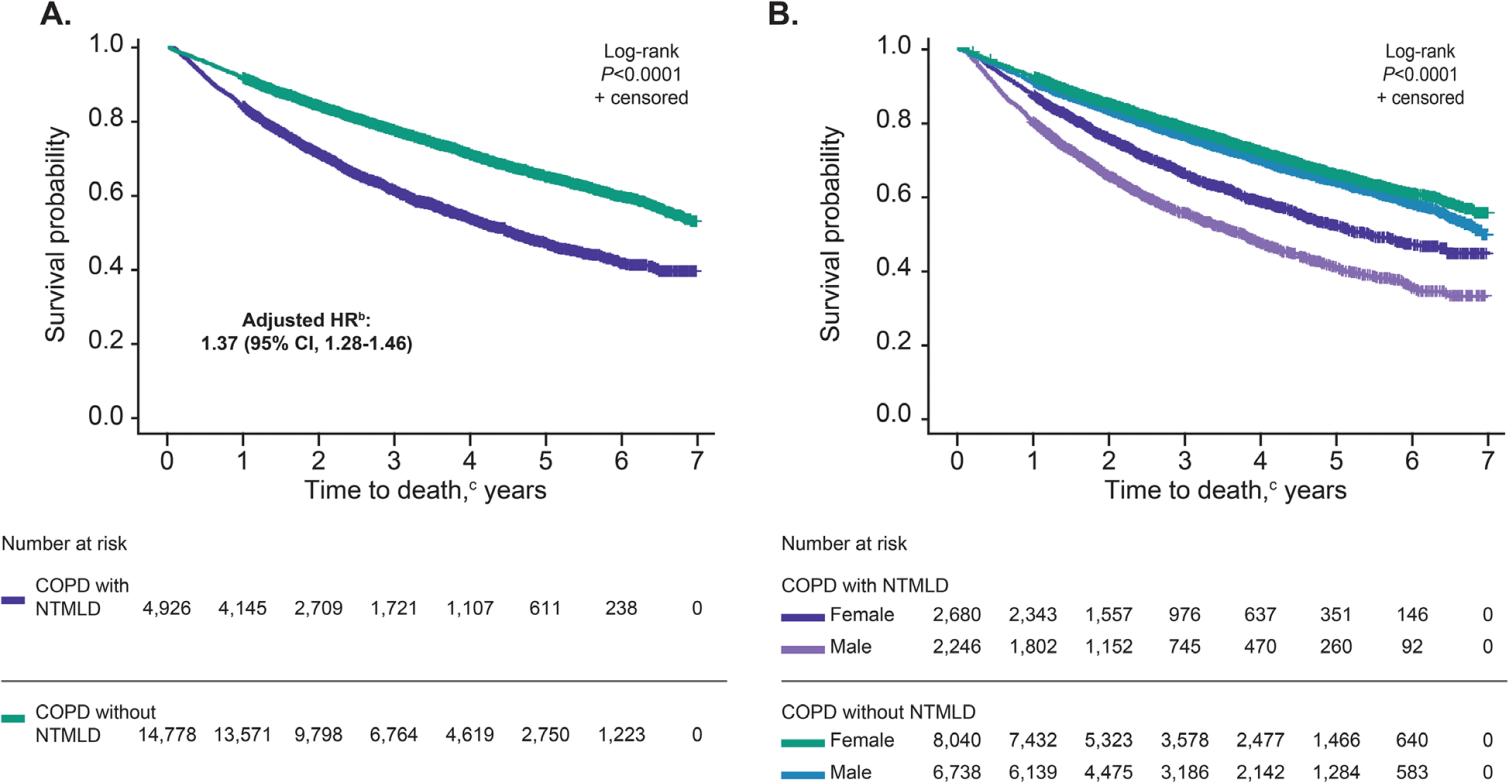
Kaplan-Meier survival probability^a^ stratified by: presence of NTMLD (**A**); sex and NTMLD (**B**). COPD, chronic obstructive pulmonary disease; HR, hazard ratio; NTMLD, nontuberculous mycobacterial lung disease. ^a^Univariate analysis. ^b^Adjusted HR associated with NTMLD was derived from Cox proportional hazards model analysis controlling for age, sex, comorbidities, and COPD severity during the 1-year pre-index period. ^c^Time to death was defined as years from index date to death or data cutoff (December 31, 2017)

In the Kaplan-Meier survival analysis, the hazard of death was higher among patients with both COPD and NTMLD than patients with COPD without NTMLD (*P* < 0.0001; Fig. 3A). Time to death was much shorter among patients with both COPD and NTMLD than control patients with COPD without NTMLD. The twenty-fifth percentile mortality occurred at 1.68 (95% CI, 1.57–1.78) years for patients with COPD and NTMLD vs. 3.40 (95% CI, 3.27–3.53) years for control patients with COPD without NTMLD. Similarly, in multivariate Cox proportional hazards model analysis adjusting for age, sex, selected comorbidities, and markers of COPD severity during the 1-year pre-index period, patients with both COPD and NTMLD had 37% higher risk of death than patients with COPD without NTMLD (HR, 1.37; 95% CI, 1.28–1.46; *P* < 0.0001).

The hazard of death was higher among male patients with COPD and NTMLD than among female patients with COPD and NTMLD, as shown in the Kaplan-Meier curve (*P* < 0.0001; Fig. 3B). After multivariate Cox proportional hazards analysis adjusting for confounding factors during the 1-year pre-index period, the HR of mortality associated with male relative to female sex was 1.23 (95% CI, 1.17–1.30; *P* < 0.0001).

## Discussion

In this retrospective matched-cohort study of US Medicare beneficiaries, patients with COPD and NTMLD had a higher mortality burden than patients with COPD without NTMLD. This finding was consistent in both univariate and multivariate analyses which included fully adjusted odds of death during follow-up (OR, 1.39; 95% CI, 1.27–1.51; *P* < 0.0001), Poisson mortality rate (RR, 1.36; 95% CI, 1.28–1.45; *P* < 0.0001), and survival analysis (HR, 1.37; 95% CI, 1.28–1.46; *P* < 0.0001).

The study found that patients with NTMLD had more severe COPD than control patients without NTMLD based on our surrogate markers of COPD severity. For example, the rates of COPD-related hospitalization, COPD-related ED visits, and supplemental oxygen use were all higher in cases than in control patients, even when stratified by survival status (alive vs. deceased) at the end of follow-up, with higher pre-index COPD severity noted among deceased patients in both groups. Although patients with both COPD and NTMLD had more severe COPD, the risk of death remained significantly higher among patients with COPD and NTMLD after controlling for COPD severity, along with age, sex, and selected comorbidities. This increased risk of death may identify an opportunity to improve outcomes through early screening for NTMLD among COPD patients.

Some studies have hypothesized that lung cancer might allow the establishment of infection with *Mycobacterium avium* complex, the most common species of nontuberculous mycobacteria in the US [41]. Alternatively, others have suspected that *Mycobacterium avium* complex lung disease may be a predisposing factor for lung cancer [42]. In our study, 16.2% of cases and 3.1% of control patients had lung cancer during the 1-year pre-index period (Table 1). We removed pre-index lung cancer in the multivariate analyses and found that there was still a statistically significant higher risk for mortality associated with NTMLD (OR, 1.50; 95% CI, 1.38–1.64; RR, 1.44; 95% CI, 1.35–1.53; HR, 1.45; 95% CI, 1.36–1.54).

Our data are consistent with the findings of a smaller-scale analysis from a German research database, which reported that a higher proportion of patients with both COPD and NTMLD died within 3.25 years of observation compared with age-, sex-, and Charlson Comorbidity Index–matched patients with COPD without NTMLD (41.5% [27/65] vs. 15.9% [62/390]; *P* < 0.001) [33]. The relationship between concurrent COPD and NTMLD in terms of mortality burden was also explored in a matched case-control study that used data from the US National Center for Health Statistics (1999–2014); NTMLD-related deaths were defined as those listing NTMLD (*ICD-10-CM*: A31.0, A31.1, A31.8, A31.9) as either the underlying or contributing cause of death [43]. In the National Center for Health Statistics–based investigation, comorbid COPD and emphysema diagnoses were strongly associated with NTMLD-related deaths (OR, 4.05; 95% CI, 3.86–4.25) compared with an age-, sex-, and race/ethnicity-matched population control [43].

In the German study that demonstrated a dramatically higher mortality among patients with COPD and NTMLD (compared with patients with COPD without NTMLD), the severity of COPD and other clinical factors was not provided [33]. An insurmountable challenge that remains, despite using statistical methods to adjust for comorbidity, is that of causal inference. Whether nontuberculous mycobacterial disease per se caused the excess deaths observed in the COPD with NTMLD cohort cannot truly be known. It is without doubt that NTMLD can be a fatal disease [44]. In a national nested case-control study of patients treated by the Veterans Health Administration, patients with NTMLD had both an increased risk of outpatient visits and an increased risk of mortality, after adjusting for COPD, structural lung diseases, and immunomodulatory factors [45]. Additionally, in a population-based study in Ontario, Canada, patients with NTMLD, carefully matched by age, sex, and propensity score, had substantially higher rates of mortality than control patients [4]. However, it is important to note that causality in case-control studies may not be as easy to assess as in other study types, such as in case series. Although the mechanism for increased mortality is unknown, we believe that there is more than one. We hypothesize that a minority of patients die directly from progression of NTMLD, while a much larger proportion experience infection-related deterioration of their health and, with the frailty of an additional disease, have reduced survival.

There are inherent limitations in retrospective claims-based epidemiologic studies, including variations in coding practices and the potential for coding inaccuracy (overcoding, undercoding, miscoding) [6, 46]. Case ascertainment methodology similar to that used in our investigation has reported a positive predictive value of 72% and a sensitivity of 42% for identifying NTMLD among patients with bronchiectasis, supporting that this method is valid for identifying NTMLD; the relatively low sensitivity suggested that NTMLD was often underdiagnosed or miscoded in claims-based epidemiologic research [35]. Another limitation is the absence of *ICD* codes designated for fibrocavitary NTMLD and chest imaging or radiographic reports that might indicate the fibrocavitary form. As such, the proportion of patients with fibrocavitary NTMLD, which has been associated with an increased mortality burden [47], in this study is unknown.

A limitation of this study is that pharmacotherapy data, which are not available in Medicare Parts A and B data, were not included to confirm the validity of the COPD patient population. The detection of patients with COPD may be more accurate when more criteria are combined [48], and including pharmacotherapy data, especially inhaled medicines for COPD, may provide additional supportive criteria to confirm COPD and estimate its severity. Considering this limitation, the confirmation of COPD in this study followed other previously published approaches using US inpatient and ambulatory claims [36, 49], including the findings from a systematic review of 38 publications that *ICD-9* or *ICD-10* coding was the most frequently used for identifying COPD patients in health systems (34 of 38 studies used *ICD* codes), followed by hospitalization/ambulatory data (22–30 studies) and drug prescription data (18 studies) [48]. Future studies in this population should be designed to include pharmacotherapy data on inhaled medicines for COPD when possible.

Another limitation in claims-based research, specific to the current analysis, is that established measures of COPD severity such as spirometry findings (i.e., forced expiratory volume in 1 s, forced vital capacity) could not be captured by claims data; thus, we developed surrogate markers of severity. Use of surrogate markers such as supplemental oxygen to characterize severity of COPD in claims-based analyses has been described previously [50]. While our study included health care utilization–based surrogate markers, Mapel and colleagues used “COPD complexity”–based pulmonary comorbidities and medical procedures as a surrogate for COPD disease severity. Given that having long-term COPD may contribute to disease severity, our severity assessment was limited in the sense that it did not capture COPD diagnosis prior to Medicare enrollment. Lastly, our analysis was based on US Medicare beneficiaries; the findings may not be generalizable to commercially insured populations or populations in other countries. Nonetheless, these analyses provide the first large-scale US population–based estimate of the mortality burden associated with NTMLD in patients with COPD.

## Conclusions

The substantial incremental mortality burden associated with NTMLD in patients with COPD highlights the importance of developing interventions targeting this high-risk group and may indicate an unmet need for timely and appropriate management of NTMLD.

### Electronic supplementary material

Below is the link to the electronic supplementary material.

**Additional File 1**. “*ICD* Diagnostic Codes and *CPT* Codes for Defining NTMLD, COPD, and Bronchiectasis”. An overview of the *ICD* diagnostic codes and *CPT* codes used for defining NTMLD, COPD, and bronchiectasis.

**Additional File 2**. “Surrogate Markers of COPD Severity”. An overview of the *ICD* diagnostic codes and *CPT* codes used for identifying surrogate markers of COPD severity.

**Additional File 3**. “*ICD* Diagnostic Codes for Comorbidities and Symptoms”. An overview of the diagnostic codes used for identifying comorbidities and symptoms.

## Data Availability

The data sets generated and/or analyzed during the current study are available in the Center for Medicare Services Virtual Research Data Center repository, https://resdac.org/.

## Abbreviations

CI: confidence interval
COPD: chronic obstructive pulmonary disease
ED: emergency department
HR: hazard ratio
*ICD-9-CM*: *International Classification of Diseases, Ninth Revision, Clinical Modification*
*ICD-10-CM*: *International Classification of Diseases, Tenth Revision, Clinical Modification*
NTMLD: nontuberculous mycobacterial lung disease
OR: odds ratio
RR: rate ratio
SD: standard deviation
US: United States
