# Supplementary material for: Incremental mortality associated with nontuberculous mycobacterial lung disease among US Medicare beneficiaries with chronic obstructive pulmonary disease
Source: BMC Infect Dis. 2023 Nov 1;23:749. doi: 10.1186/s12879-023-08689-9 (PMC10619258; doi:10.1186/s12879-023-08689-9)
Supplement: Supplementary file 1 — Additional File 1. “ICD Diagnostic Codes and CPT Codes for Defining NTMLD, COPD, and Bronchiectasis”. An overview of the ICD diagnostic codes and CPT codes used for defining NTMLD, COPD, and bronchiectasis. [file 12879_2023_8689_MOESM1_ESM.docx]

**Additional File 1. ICD Diagnostic Codes and CPT Codes for Defining NTMLD, COPD, and Bronchiectasis**

| Diagnostic Codes | ICD-9-CM^a^ | ICD-10-CM^a^ |
| --- | --- | --- |
| NTMLD | 031.0 | A31.0 |
| COPD | 491.0, 491.1, 491.8, 491.9, 491.20, 491.21, 491.22, 492.0, 492.8, 496 | J41.0, J41.1, J41.8, J42, J43.0, J43.1, J43.2, J43.8, J43.9, J44.0, J44.1, J44.9 |
| Bronchiectasis^b^ | 494.0, 494.1 | J47.0, J47.1, J47.9 |
|  | | |
| Codes for chest CT | CPT | Description |
|  | 71250 | CT of thorax; without contract material |
|  | 71260 | CT of thorax, with contrast material(s) |
|  | 71270 | CT of thorax; without contrast material, followed by contrast material(s) and further sections |

COPD, chronic obstructive pulmonary disease; CPT, Current Procedural Terminology; CT, computed tomography; ICD-9-CM, *International Classification of Diseases, Ninth Revision, Clinical Modification*; ICD-10-CM, *International Classification of Diseases, Tenth Revision, Clinical Modification;* NTMLD, nontuberculous mycobacterial lung disease.

^a^ICD-9-CM codes were used for claims dated prior to October 1, 2015; ICD-10-CM codes were used for claims dates after October 1, 2015.

^b^Patients with bronchiectasis were excluded in this study.

A patient with bronchiectasis was defined as a beneficiary who had either ≥2 ambulatory encounters with a diagnostic code for bronchiectasis (see above **Table**) that were dated ≥30 days apart, ≥1 ambulatory encounter with a bronchiectasis diagnosis and a computed tomography scan of the thorax within 60 days prior to the encounter, or ≥1 hospitalizations with a principal or secondary diagnosis for bronchiectasis.(1)

Reference

1. Seifer FD, Hansen G, Weycker D. Health-care utilization and expenditures among patients with comorbid bronchiectasis and chronic obstructive pulmonary disease in US clinical practice. Chron Respir Dis. 2019;16:1479973119839961.
