# Supplementary material for: Incremental mortality associated with nontuberculous mycobacterial lung disease among US Medicare beneficiaries with chronic obstructive pulmonary disease
Source: BMC Infect Dis. 2023 Nov 1;23:749. doi: 10.1186/s12879-023-08689-9 (PMC10619258; doi:10.1186/s12879-023-08689-9)
Supplement: Supplementary file 2 — Additional File 2. “Surrogate Markers of COPD Severity”. An overview of the ICD diagnostic codes and CPT codes used for identifying surrogate markers of COPD severity. [file 12879_2023_8689_MOESM2_ESM.docx]

**Additional File 2. Surrogate Markers of COPD Severity**

| Surrogate markers of COPD severity   - Supplemental oxygen use   - Home oxygen therapy was identified using HCPCS codes E0424, E0431, E0433, E0434, E0439, E0441, E0442, E0443, E0444, E1390, E1391, E1392, or K0738 in durable medical equipment claims   - Dependence on supplemental oxygen was identified using ICD-9-CM V46.2 or ICD-10-CM Z99.81 in any position in ambulatory (carrier or outpatient claims) or inpatient claims - Pulmonary function tests   - Identified using CPT codes for spirometry 94010 or 94060 in ambulatory or inpatient claims - Duration from COPD diagnosis to index date (ie, date of first NTMLD diagnosis)   - COPD diagnosis date was the date of first claim of COPD that fulfilled the COPD definition - COPD-related hospitalizations   - Identified using COPD ICD-9-CM and ICD-10-CM codes (e-Table 1) as principal or secondary diagnosis from inpatient claims - COPD-related ED visits   - Identified using COPD ICD-9-CM and ICD-10-CM codes (e-Table 1) as principal or secondary diagnosis from both inpatient and outpatient claims with indicators for ED visits |
| --- |

COPD, chronic obstructive pulmonary disease; CPT, Current Procedural Terminology; ED, emergency department; HCPCS, Healthcare Common Procedure Coding System.
