# Supplementary material for: Incremental mortality associated with nontuberculous mycobacterial lung disease among US Medicare beneficiaries with chronic obstructive pulmonary disease
Source: BMC Infect Dis. 2023 Nov 1;23:749. doi: 10.1186/s12879-023-08689-9 (PMC10619258; doi:10.1186/s12879-023-08689-9)
Supplement: Supplementary file 3 — Additional File 3. “ICD Diagnostic Codes for Comorbidities and Symptoms”. An overview of the diagnostic codes used for identifying comorbidities and symptoms. [file 12879_2023_8689_MOESM3_ESM.docx]

**Additional File 3. ICD Diagnostic Codes for Comorbidities and Symptoms**

| Comorbidities | ICD-9-CM | ICD-10-CM |
| --- | --- | --- |
| Nonpulmonary comorbid conditions | | |
| All cancers, excluding lung cancer | 140-161, 163-209 | C00-C33, C37-C96 |
| All cardiovascular diseases | 390-459 | I00-I99 |
| Chronic kidney disease | 585 | N18 |
| Chronic viral hepatitis | 07032, 07033, 07044, 07054 | B18 |
| Crohn’s disease | 555 | K50 |
| Dementia | 290, 291, 292, 294, 331, 332 | F00, F01, F02, F03, F05, G30, G31, G20 |
| Diabetes mellitus | 250 | E10, E11 |
| Gastroesophageal reflux disease | 53081, 53011 | K21 |
| Human immunodeficiency virus | 042 | B20 |
| Hypertension | 401-405 | I10-I15 |
| Malnutrition | 260-269 | E40-E46 |
| Moderate or severe liver disease | 4560, 4561, 45620, 45621, 5722, 5723, 5724, 5728 | K704, K711, K721, K729, K765, K766, K767, I850, I859, I864, I982 |
| Multiple sclerosis | 340 | G35 |
| Overweight and obesity | 2780 | E66 |
| Rheumatoid arthritis | 714 | M05 |
| Systemic lupus erythematosus | 7100 | M321, M328, M329 |
| Transplant of kidney, heart, or liver | V420, V421, V427 | Z940, Z941, Z944 |
| Ulcerative colitis | 556 | K51 |
| Underweight or abnormal weight loss | 78321, 78322 | R634, R636 |
| Pulmonary symptoms | | |
| Cough | 7862 | R05 |
| Dyspnea | 78602, 78605, 78609 | R060 |
| Hemoptysis | 7863 | R04 |
| Pulmonary comorbidities | | |
| Asthma | 493 | J45 |
| Cystic fibrosis with pulmonary manifestations | 27702 | E840 |
| Emphysema | 492 | J43 |
| Idiopathic interstitial lung disease | 515, 5160, 5161, 5162, 5163, 5164, 5165, 51661, 51662, 51663, 51664, 51669, 5169 | J8410, J8489, J8401, J8403, J8402, J84111, J84112, J84113, J84114, J84115, J842, J84116, J84117, J8481, J8482, J84841, J84842, J8483, J84843, J84848, J849 |
| Idiopathic pulmonary fibrosis | 51631 | J84112 |
| Lung transplant | V426 | Z942, Z943 |
| Malignant neoplasm of bronchus and lung | 162 | C34 |
| Pneumonia | 480-486 | J12-J18 |
| Pulmonary tuberculosis | 011 | A15 |
| Simple and mucopurulent chronic bronchitis | 491 | J41 |
| Smoking history | V1582 | Z87891 |

Comorbidities were defined by the presence of a single ICD code in any position from fee-for-service institutional inpatient, outpatient, or noninstitutional carrier claims.

ICD-9-CM, *International Classification of Diseases, Ninth Revision, Clinical Modification*; ICD-10-CM, *International Classification of Diseases, Tenth Revision, Clinical Modification.*
